# Human AI Teaming for Coronary CT Angiography Assessment: Impact on Imaging Workflow and Diagnostic Accuracy

**DOI:** 10.3390/diagnostics13233574

**Published:** 2023-11-30

**Authors:** Florian Andre, Philipp Fortner, Matthias Aurich, Sebastian Seitz, Ann-Kathrin Jatsch, Max Schöbinger, Michael Wels, Martin Kraus, Mehmet Akif Gülsün, Norbert Frey, Andre Sommer, Johannes Görich, Sebastian J. Buss

**Affiliations:** 1 Department of Cardiology, Angiology and Pneumology, University of Heidelberg, 69120 Heidelberg, Germany; norbert.frey@med.uni-heidelberg.de; 2 MVZ DRZ Heidelberg, 69126 Heidelberg, Germany; dr.fortner@mvz-drz.de (P.F.); dr.aurich@mvz-drz.de (M.A.); dr.seitz@mvz-drz.de (S.S.); prof.buss@mvz-drz.de (S.J.B.); 3 Siemens Healthineers, 91301 Forchheim, Germany; max.schoebinger@siemens-healthineers.com (M.S.); martin_kraus@siemens-healthineers.com (M.K.); 4 Siemens Healthineers, Princeton, NJ 08540, USA

**Keywords:** coronary artery disease, coronary CT angiography, artificial intelligence, workflow, human AI teaming, computed tomography

## Abstract

As the number of coronary computed tomography angiography (CTA) examinations is expected to increase, technologies to optimize the imaging workflow are of great interest. The aim of this study was to investigate the potential of artificial intelligence (AI) to improve clinical workflow and diagnostic accuracy in high-volume cardiac imaging centers. A total of 120 patients (79 men; 62.4 (55.0–72.7) years; 26.7 (24.9–30.3) kg/m^2^) undergoing coronary CTA were randomly assigned to a standard or an AI-based (human AI) coronary analysis group. Severity of coronary artery disease was graded according to CAD-RADS. Initial reports were reviewed and changes were classified. Both groups were similar with regard to age, sex, body mass index, heart rate, Agatston score, and CAD-RADS. The time for coronary CTA assessment (142.5 (106.5–215.0) s vs. 195.0 (146.0–265.5) s; *p* < 0.002) and the total reporting time (274.0 (208.0–377.0) s vs. 350 (264.0–445.5) s; *p* < 0.02) were lower in the human AI than in the standard group. The number of cases with no, minor, or CAD-RADS relevant changes did not differ significantly between groups (52, 7, 1 vs. 50, 8, 2; *p* = 0.80). AI-based analysis significantly improves clinical workflow, even in a specialized high-volume setting, by reducing CTA analysis and overall reporting time without compromising diagnostic accuracy.

## 1. Introduction

Cardiovascular disease is the leading cause of death worldwide and has increased over the past decade, posing a significant challenge to public health systems [1]. Strategies for early detection of disease and risk stratification of patients are of great clinical and socioeconomic interest. Coronary computed tomography angiography (CTA) has emerged as an important diagnostic modality in the assessment of patients with stable chest pain and is being implemented in several national and international guidelines [2,3,4].

Coronary CTA offers excellent periprocedural safety and risk stratification equivalent to invasive coronary angiography [5,6]. As coronary CTA allows for the detection of early and nonobstructive stages of coronary artery disease (CAD) and for the identification of high-risk plaques, it enables the timely initiation of adequate preventive therapies [7,8]. The increasing use of coronary CTA is associated with fewer hospitalizations for myocardial infarction and a reduction in cardiovascular mortality [9]. The detailed assessment of the entire coronary tree, including stenosis quantification and plaque characterization, requires both time and expertise. With the increasing use of coronary CTA and the associated increase in workload, technologies that optimize workflow and improve diagnostic accuracy are of great interest. Artificial intelligence (AI) has been adopted for coronary CTA assessment showing promising results, but improvements in the integration of AI-based applications into clinical workflow are still required [10,11].

Recently, AI-based coronary CTA analysis software has been introduced that provides an automated pre-evaluation of the entire coronary tree in terms of luminal narrowing and plaque composition. In contrast to other available approaches, it allows for the on-site processing and evaluation of the CTA series [12,13], thereby enabling a seamless integration into the clinical workflow. Thus, it may facilitate and speed up the diagnostic workflow as well as provide additional diagnostic confidence by serving as a second reader.

The objective of the study was to investigate the potential of AI for improving clinical workflow and diagnostic accuracy.

## 2. Materials and Methods

### 2.1. Image Acquisition

Patients, who were referred for coronary CTA by their physician, were assigned to either a standard assessment group or to an AI-based assessment group (human AI team) using block randomization. Patients with contraindications to contrast medium application, prior percutaneous coronary interventions, or coronary artery bypass grafts were not included in the study since the AI has yet not been trained for the analysis of such cases. Image acquisition was performed on third generation a dual-source CT scanner (SOMATOM Force, Siemens Healthcare, Forchheim, Germany) in a high-volume imaging center performing >2500 coronary CTA examinations per year. Patients received up to 20 mg metoprolol tartrate intravenously to achieve a heart rate of approximately 65/min and 0.4 mg glyceroltrinitrate if no contraindications were present. A calcium scoring scan was used to determine the coronary calcium burden and to further optimize the subsequent coronary CTA protocol. Depending on heart rate, heart rhythm, and calcium burden, axial or spiral acquisition modes were used for coronary CTA. Images were reconstructed using Advanced Modeled Iterative Reconstruction (ADMIRE) level 3 with dedicated cardiac kernels (usually Bv36 and Bv40). The series of the automatically determined best diastolic (best diastole) or best systolic (best systole) phase was automatically transferred to the on-site AI server and processed without any user interaction.

### 2.2. Image Analysis

**Figure 1:**
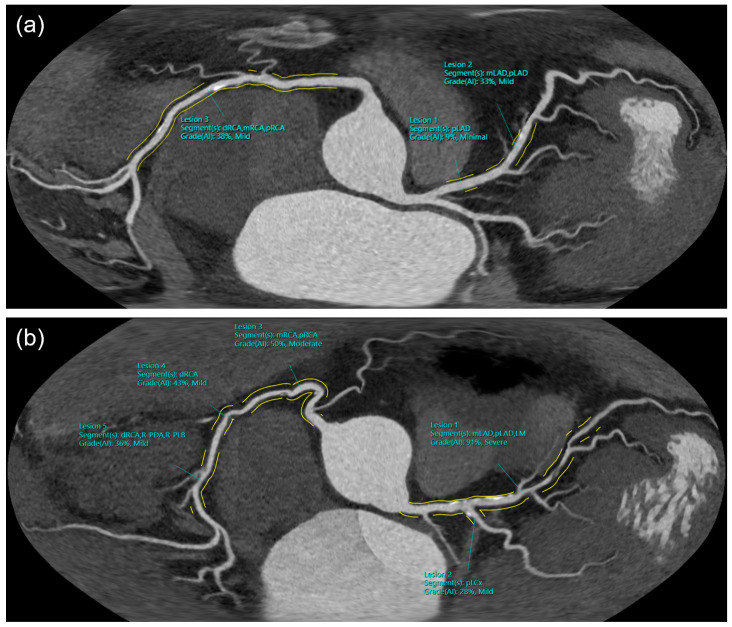
Examples of the unfolded view. (**a**) patient with mild CAD (CAD-RADS 2) and (**b**) patient with severe CAD (CAD-RADS 4A).

Image analysis was performed on a dedicated workstation (syngo.via VB60, Siemens Healthcare, Forchheim, Germany) by two readers with >5 years and >10 years of experience in cardiac CT. A dedicated on-site database with a browser-based interface was used for structured reporting. The results of the coronary artery assessment were recorded in a table containing stenosis severity and plaque composition of every segment of the coronary tree model recommended by the Society of Cardiovascular Computed Tomography (SCCT) [14,15]. In the standard group, the reader analyzed every coronary artery segment visually and performed semi-automated stenosis measurements as needed. The results were manually entered into the table, which was facilitated by providing drop-down menus and boilerplates. In the human AI group, the results of the fully automated analysis were transferred to the table automatically and an unfolded view of the coronary tree was provided as an overview of the analysis results (Figure 1). The reader needed to review the results of the AI and correct them, if necessary. In both groups, the time taken from the start of the coronary artery analysis to the completion of the table was measured (coronary CTA assessment time). Subsequently, the cardiac morphology was assessed and a structured report, which included the analysis of the coronary arteries using the Coronary Artery Disease Reporting and Data System (CAD-RADS), other cardiac findings (e.g., atrial septal defects), and the resulting recommendations, was created. The time between the start of the coronary artery analysis and the finalization of the entire report was measured (total reporting time). The readers rated their diagnostic confidence on a scale of 1 (low) to 5 (high). The initial report was reviewed by a third reader (>15 years of experience in cardiac CT), and a consensus reading was performed in case of discrepancies. Changes from the original report were considered minor, if the CAD-RADS score had not changed, or relevant, if it had. As the AI was not trained to detect intramural plaques, CAD-RADS scores 0 and 1 were combined into one group.

### 2.3. AI Analysis

Automated analysis of the coronary CTA series was performed using a non-commercial research prototype (Siemens Automatic Coronary Analysis Prototype 2.0, Siemens Healthcare, Forchheim, Germany). The software provides a fully automated workup of the case in four steps. In the first step, the patient-specific coronary anatomy is automatically segmented using the approach described in Gülsün et al. [16]. This approach first performs four-chamber segmentation and detects key cardiac landmarks, including the mitral valve center, aortic valve center, and left-ventricular apex. It then detects the left and right coronary ostia, followed by automatic extraction of the entire coronary artery centerline tree. The centerline extraction algorithm uses model-based coronary specific territories and main branches for robustness against stents, imaging artifacts, and total occlusions.

In the second step, the SCCT 18-segment model is applied to the coronary artery branches using an automated coronary labeling approach that includes a 3D image-to-image deep learning model and a post-processing step to enforce anatomical consistency of the labeling results. The 3D deep learning model is trained to predict a segment label class for each coronary voxel, given an input intensity mask that encodes the four heart chambers and the coronary tree. The architecture consists of encoder and decoder blocks, predicting a class from a comprehensive segment-label set of 26 coronary segments. Each centerline point is then assigned a label based on the prediction of the closest voxel. The model is trained on an independent set of 3914 cases with expert-annotated segment labels, of which 628 were used as a separate validation set for the final model selection. The post-processing step takes the coronary centerline trees with deep learning generated label predictions as input and finds a maximum likelihood solution according to a set of rules that define all possible parent child label configurations.

In the third step, the actual vessel assessment is performed. A multitask deep learning approach is applied to perform automated coronary lesion detection, lumen segmentation, and healthy vessel wall estimation. The approach is trained for multiple tasks to account for potential confounding factors such as poor image quality or the presence of other causes of stenosis (e.g., myocardial bridging). The multitask deep learning architecture is trained jointly for improved generalization performance and robustness of the individual tasks. The decoder for each anomaly detection task predicts a probability distribution over output classes (such as healthy vs. diseased) for each centerline point. The decoder for segmentation tasks predicts a probability distribution over output segmentation classes (foreground vs. background) for each voxel. Cross-sectional images with an isotropic resolution of 0.2 mm and a size of 41 × 41 are sampled along the coronary centerline and stacked to construct a 3D input passed to the model. The model is trained on a separate set of 3491 cases with expert-annotated lesion, stent, myocardial-bridging and artifact markers, and segmentation masks where 818 of them are used for the final model selection. A fixed probability threshold (0.5) is applied to the model’s outputs to obtain final marker positions and segmentation masks.

**Figure 2:**
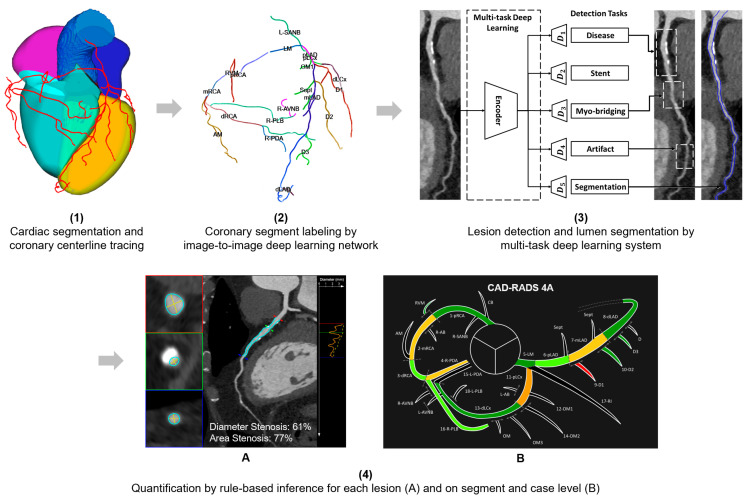
Visualization of the AI analysis workflow after segmentation of the coronary centerlines: (**1**) a coronary segment labeling is performed; (**2**) after lesion detection and lumen segmentation; (**3**) quantification is performed on lesion-, segment- and case-level; (**4**) quantification by rule-based inference for each lesion (**4A**) and on segment and case level (**4B**).

In a fourth step, quantification is performed at lesion-, segment-, and case-level by a rule-based inference mechanism. Stenosis grading is performed for each lesion by comparing the minimum luminal diameter to the reference diameter derived from the healthy vessel wall segmentation. The maximum degree of narrowing is determined for each coronary segment by computing the maximum degree of narrowing of all lesions present in that segment. Finally, the CAD-RADS score is calculated based on the maximum degree of stenosis across all evaluated segments. The results of the analysis are presented to the user with overview images as well as lesion-specific key images. An overview of the workflow is shown in Figure 2. The overview images provide a generalized, color-coded schematic depiction of the coronary segments as well as a coronary unfolded view showing the detected lesions in relation to the patient’s individual coronary anatomy (Figure 1). The quantitative results were stored as structured information and provided to the reporting system in JavaScript Object Notation (JSON) format through a software interface, which was used for the automated transfer of the results of the AI into the table of the reporting database in this study.

Patient characteristics were derived from medical records and questionnaires. The study was conducted in accordance with the Declaration of Helsinki and the scientific data analysis was approved by the local ethics committee (S-758/2018).

### 2.4. Statistics

Data was assessed for normal distribution using the D’Agostino–Pearson test. Categorial data is given as counts and fractions and continuous data is presented uniformly as median and interquartile range since it showed partly a non-parametric distribution. The Mann–Whitney test was applied for the comparison of continuous data and the Chi-squared test for categorical data. Statistical analyses were performed using dedicated statistical software (MedCalc Statistical Software Version 22, MedCalc Software, Ostend, Belgium). A *p*-value < 0.05 was regarded as statistically significant.

## 3. Results

### 3.1. Patient Characteristics

**Figure 3:**
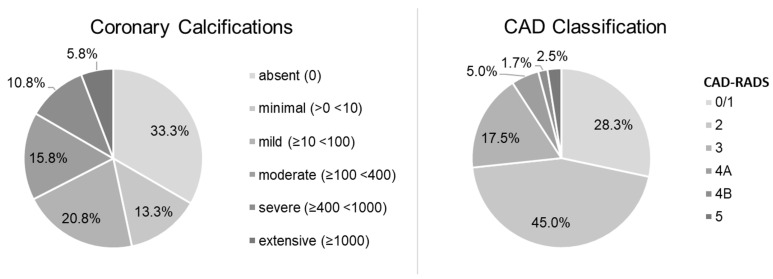
Distribution of coronary calcifications and CAD severity in the study population.

The study population consisted of 120 patients (79 men, 41 women) with a median age of 62.4 (55.0–72.7) years. Men were significantly younger than women (61.4 (54.5–69.4) years vs. 66.9 (55.5–75.7) years; *p* < 0.05). The median body mass index (BMI) was 26.7 (24.9–30.3) kg/m^2^ and the median heart rate during the coronary CTA was 61.5 (57.5–65.5)/min. The median Agatston score was 20.1 (0.0–174.6) and coronary calcifications were absent in 40 patients (33.3%), minimal in 16 patients (13.3%), mild in 25 patients (20.8%), moderate in 19 patients (15.8%), severe in 13 patients (10.8%), and extensive in 7 patients (5.8%). The CAD severity classified by CAD-RADS was as follows: CAD-RADS 0/1 in 34 patients (28.3%), CAD-RADS 2 in 54 patients (45.0%), CAD-RADS 3 in 21 patients (17.5%), CAD-RADS 4A in 6 patients (5.0%), CAD-RADS 4B in 2 patients (1.7%), and CAD-RADS 5 in 3 patients (2.5%). The distribution of coronary calcifications and CAD severity is displayed in Figure 3.

The renal function and the distribution of cardiovascular risk factors other than smoking within the past 5 years were not significantly different between the standard and human AI group (see Table 1). Of note, both groups showed no significant differences with regard to age, sex, BMI, heart rate, Agatston score, and CAD-RADS resulting in comparable conditions for the coronary artery analysis.

### 3.2. Workflow Optimization

**Figure 4:**
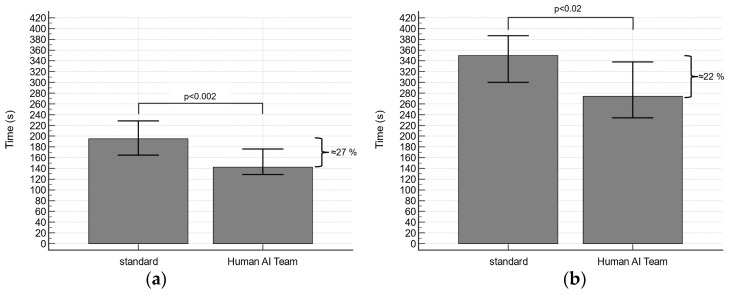
The use of AI resulted in a significant reduction in both (**a**) coronary CTA assessment time and (**b**) total reporting time. Median with 95% confidence interval.

The time for the coronary CTA assessment was significantly reduced in the human AI group compared to the standard group by approximately 27% (142.5 (106.5–215.0) s vs. 195.0 (146.0–265.5) s; *p* < 0.002; Figure 4a). Accordingly, the total reporting time was significantly lower in the human AI group (274.0 (208.0–377.0) s vs. 350.0 (264.0–445.5) s; *p* < 0.02; Figure 4b), which corresponds to approximately 22%.

**Figure 5:**
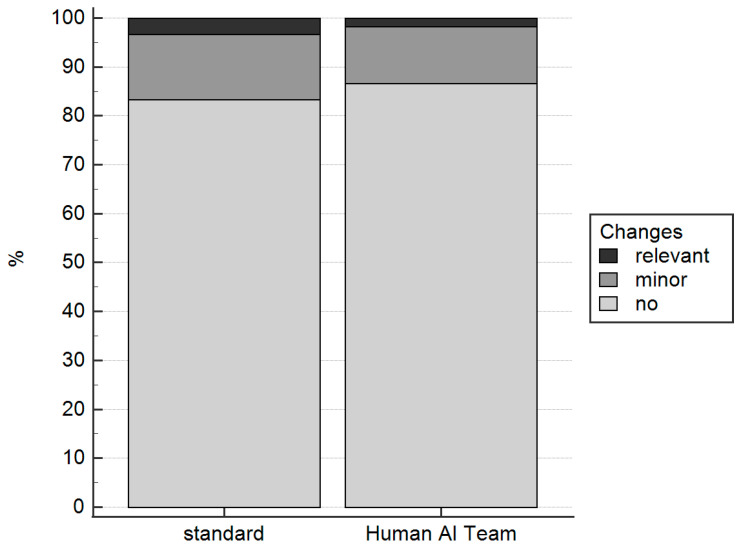
The number of cases with no changes, with minor changes, or with changes relevant to CAD-RADS did not differ significantly between the standard group and the human AI group.

Diagnostic certainty was comparable between the human AI and the standard group (4.0 (4.0–5.0) vs. 4.0 (3.0–5.0); *p* = 0.52). The number of cases with no, minor, or CAD-RADS relevant changes did not differ significantly between the human AI and the standard group (52, 7, 1 vs. 50, 8, 2; *p* = 0.80; Figure 5).

## 4. Discussion

This is the first study to assess this recently introduced AI for coronary CTA analysis that has been fully integrated into the workflow under clinical conditions in a real-world population. Its use resulted in a significant reduction in coronary CTA assessment time and total reporting time, without compromising diagnostic accuracy.

As coronary CTA is increasingly included in guidelines and is likely to become the first-line modality in the evaluation of patients with stable chest pain in an increasing number of health systems, it can be assumed than examination numbers will increase. With the associated significant increase in workload, there is a high demand for automation and optimization throughout the imaging workflow [10]. AI approaches have the potential to address this need by improving image reconstruction, including image reformation, and automating coronary CTA analysis. Adequate visualization of the coronary tree, as provided by the unfolded view of the AI (Figure 1) may not only facilitate the assessment of CAD, but also allow for the demonstration of findings to patients.

Previous studies have assessed several AI applications for coronary artery assessment. In a retrospective multicenter study including 527 patients, a fully automated AI algorithm was not inferior to experts in the detection of coronary stenosis ≥50% and reduced the post-processing time significantly [17]. An accuracy of 84% and 86% for detecting ≥50% and ≥70% stenoses, respectively, was found in a CREDENCE substudy comparing AI quantitative coronary CTA with core lab quantitative coronary angiography [18]. In the CLARIFY study including 232 patients, there was high agreement between the AI approach and experienced readers for CAD-RADS classification, with 78.0% of cases achieving CAD-RADS categorical agreement and 98.3% of cases agreeing within one category [12]. Accordingly, the results of the human AI team and those of the reference reading showed a good agreement in our study.

While many of the previous studies took a technical approach, focusing on the accuracy of the respective AI approaches, the scope of this study was to evaluate the potential of an AI-based approach to optimize the clinical workflow. For example, the cardiac phase was automatically selected by the CT scanner software and not meticulously chosen with regard to most appropriate image quality. This approach may not fully assess the diagnostic accuracy of AI as it had also to cope with series of suboptimal cardiac phases. However, this approach may give a more realistic impression of the current clinical potential of AI, as a fully autonomous coronary CTA analysis without human interaction and supervision seems unlikely within the next few years due to the current limitations of AI and regulatory requirements. Human readers will still need to review AI results and take responsibility for the final report and resulting recommendations. In particular, it will remain the physician’s task to integrate the results of the coronary CTA examination into the individual clinical context. Therefore, AI is more likely to support than replace the expert in the coming years taking the role of a second reader. Hence, the Good Machine Learning Practice for Medical Device Development guiding principles identified by the US Food and Drug Administration (FDA), Health Canada, and the United Kingdom’s Medicines and Healthcare products Regulatory Agency (MHRA) include as a guiding principle that the focus should be on the performance of the human AI team, rather than the performance of the model in isolation [19]. In this study, the human AI team performed well, significantly reducing the time required for coronary CTA analysis and complete reporting. Even in this setting, with an already streamlined workflow and highly experienced readers specialized in coronary CTA, time savings of 27% were achieved for coronary tree analysis. This significant time saving can not only allow doctors to assess more examinations, but also to invest more time in the analysis of complex cases or in the interaction with the patients. Despite the known benefits of medical therapy in reducing cardiovascular risk and improving outcome, a significant proportion of CAD patients do not adhere to their medication regimen [20,21]. As the visualization of coronary findings can improve the patient’s therapy adherence [22,23], an optimized workflow that leaves more time to show the findings and discuss results and implications with the patients could be beneficial. In this study, the demonstration of the findings was further facilitated by the automatically generated unfolded view, which clearly displays the overall extent of the coronary plaques and thus the severity of the CAD.

By acting as a second reader, preparing the report and highlighting any significant stenosis, the AI could also reduce the workload on the human reader, preventing exhaustion, especially in high-volume settings. As the AI was seamlessly integrated into the imaging workflow, working in the background, the only user interaction required was the push of a button to import the results and the unfolded view into the reporting database. The main tasks of the human reader were therefore to validate the AI-generated results by cross-checking them with the coronary CTA series, to check for other cardiac findings, and to make clinical recommendations. By not imposing additional work on the reader, the long-term acceptance of this approach is high, making human AI teaming suitable for everyday practice. Notably, neither reader confidence nor diagnostic accuracy was compromised by the accelerated diagnostic process. This is of paramount importance since coronary CTA is used for initial diagnostics and risk stratification as well as for the clarification of inconclusive preceding stress tests, both of which require a high degree of diagnostic certainty.

In the human AI team, a CAD-RADS relevant deviation occurred in a single case, resulting in a change of one CAD-RADS category. The human AI team was therefore on a par with the standard CTA assessment in terms of diagnostic accuracy. In the light of the expected increase in the number of coronary CTA examinations and the associated growing number of coronary CTA readers, AI could be an advantage, especially for inexperienced readers, who can benefit significantly from AI assistance [24]. In addition to facilitating the quantification of coronary artery stenosis and the characterization of plaque, AI can also alert them to relevant findings and thus provide an additional layer of diagnostic safety. Prospectively, AI may increase the comparability between reports from different readers or different imaging sites, which might contribute to further standardization of reporting and risk stratification.

### Limitations

Patients with coronary artery stents or coronary artery bypass grafts were excluded, as the AI was not trained for these cases. Furthermore, cases with severely impaired image quality, e.g., due to severe arrhythmias requiring multi-phase assessment, were not included as the current AI model only allows for single-phase analysis. As the study was conducted in an outpatient setting, emergency patients e.g., patients with acute coronary syndrome and unstable patients, were not included in the study population. The number of cases with CAD-RADS relevant changes was slightly lower in the human AI group than in the standard group. Although a larger sample size might have led to statistical significance, the clinical relevance of such a small effect is questionable. The readers were highly experienced, which may have led to an underestimation of the effect that the use of AI by inexperienced readers would have had on time savings and diagnostic accuracy.

## 5. Conclusions

Human AI teaming with AI-based coronary CTA analysis significantly improves clinical workflow, even in a specialized high-volume setting. Coronary CTA analysis and overall reporting time were significantly reduced without compromising diagnostic accuracy.

## Figures and Tables

**Table 1:** Characteristics of the study population.

	Standard	Human AI Team	
Age (years)	62.5 (53.8–73.3)	62.1 (55.3–71.8)	*p* = 0.63
Sex (male)	41 (68.3%)	38 (63.3%)	*p* = 0.57
BMI (kg/m^2^)	27.8 (25.2–30.4)	26.5 (24.7–30.0)	*p* = 0.49
GFR (mL/min/1.73 m^2^)	84.6 (75.0–94.0)	79.4 (73.0–89.3)	*p* = 0.08
Arterial Hypertension	34 (56.7%)	36 (60.0%)	*p* = 0.71
Hyperlipidemia	28 (46.7%)	37 (61.7%)	*p* = 0.10
Diabetes mellitus	7 (11.7%)	9 (15.0%)	*p* = 0.59
Smoking	10 (16.7%)	3 (5.0%)	*p* = 0.04
Family History of CAD	36 (60.0%)	33 (55.0%)	*p* = 0.58
Heart Rate (/min)	62.0 (58.5–65.0)	60.0 (55.0–66.5)	*p* = 0.25
Agatston Score	17.7 (0.0–128.1)	43.2 (0.0–212.6)	*p* = 0.60
CAD-RADS			*p* = 0.87
0/1	17 (28.3%)	17 (28.3%)	
2	25 (41.7%)	29 (48.3%)	
3	13 (21.7%)	8 (13.3%)	
4A	3 (5.0%)	3 (5.0%)	
4B	1 (1.7%)	1 (1.7%)	
5	1 (1.7%)	2 (3.3%)	

BMI: body mass index, CAD: coronary artery disease; GFR: glomerular filtration rate.

## Data Availability

The data presented in this study are available on request from the corresponding author. The data are not publicly available due to data protection regulations.

## References

[1] WHO The Top 10 Causes of Death. https://www.who.int/news-room/fact-sheets/detail/the-top-10-causes-of-death.

[2] Knuuti J., Wijns W., Saraste A., Capodanno D., Barbato E., Funck-Brentano C., Prescott E., Storey R.F., Deaton C., Cuisset T. (2020). 2019 ESC Guidelines for the diagnosis and management of chronic coronary syndromes. Eur. Heart J..

[3] National Institute for Health and Care Excellence (2016). Recent-Onset Chest Pain of Suspected Cardiac Origin: Assessment and Diagnosis.

[4] Gulati M., Levy P.D., Mukherjee D., Amsterdam E., Bhatt D.L., Birtcher K.K., Blankstein R., Boyd J., Bullock-Palmer R.P., Conejo T. (2021). 2021 AHA/ACC/ASE/CHEST/SAEM/SCCT/SCMR Guideline for the Evaluation and Diagnosis of Chest Pain: A Report of the American College of Cardiology/American Heart Association Joint Committee on Clinical Practice Guidelines. Circulation.

[5] Maurovich-Horvat P., Bosserdt M., Kofoed K.F., Rieckmann N., Benedek T., Donnelly P., Rodriguez-Palomares J., Erglis A., Stechovsky C., DISCHARGE Trial Group (2022). CT or Invasive Coronary Angiography in Stable Chest Pain. N. Engl. J. Med..

[6] Andre F., Fortner P., Emami M., Seitz S., Brado M., Guckel F., Sokiranski R., Sommer A., Frey N., Gorich J. (2022). Factors influencing the safety of outpatient coronary CT angiography: A clinical registry study. BMJ Open.

[7] Williams M.C., Moss A.J., Dweck M., Adamson P.D., Alam S., Hunter A., Shah A.S.V., Pawade T., Weir-McCall J.R., Roditi G. (2019). Coronary Artery Plaque Characteristics Associated with Adverse Outcomes in the SCOT-HEART Study. J. Am. Coll. Cardiol..

[8] Ovrehus K.A., Diederichsen A., Grove E.L., Steffensen F.H., Mortensen M.B., Jensen J.M., Mickley H., Nielsen L.H., Busk M., Sand N.P.R. (2021). Reduction of Myocardial Infarction and All-Cause Mortality Associated to Statins in Patients without Obstructive CAD. JACC Cardiovasc. Imaging.

[9] Weir-McCall J.R., Williams M.C., Shah A.S.V., Roditi G., Rudd J.H.F., Newby D.E., Nicol E.D. (2023). National Trends in Coronary Artery Disease Imaging. JACC Cardiovasc. Imaging.

[10] Baessler B., Gotz M., Antoniades C., Heidenreich J.F., Leiner T., Beer M. (2023). Artificial intelligence in coronary computed tomography angiography: Demands and solutions from a clinical perspective. Front. Cardiovasc. Med..

[11] Liao J., Huang L., Qu M., Chen B., Wang G. (2022). Artificial Intelligence in Coronary CT Angiography: Current Status and Future Prospects. Front. Cardiovasc. Med..

[12] Choi A.D., Marques H., Kumar V., Griffin W.F., Rahban H., Karlsberg R.P., Zeman R.K., Katz R.J., Earls J.P. (2021). CT Evaluation by Artificial Intelligence for Atherosclerosis, Stenosis and Vascular Morphology (CLARIFY): A Multi-center, international study. J. Cardiovasc. Comput. Tomogr..

[13] Khasanova E., Indraratna P., Miranda P., Takagi H., Chuang M.-y., Park K.-H., Sellers S., Leipsic J. (2022). Head to Head comparison reproducibility and inter-reader agreement of an AI based coronary stenosis algorithm vs level 3 readers. J. Cardiovasc. Comput. Tomogr..

[14] Leipsic J., Abbara S., Achenbach S., Cury R., Earls J.P., Mancini G.J., Nieman K., Pontone G., Raff G.L. (2014). SCCT guidelines for the interpretation and reporting of coronary CT angiography: A report of the Society of Cardiovascular Computed Tomography Guidelines Committee. J. Cardiovasc. Comput. Tomogr..

[15] Austen W.G., Edwards J.E., Frye R.L., Gensini G.G., Gott V.L., Griffith L.S., McGoon D.C., Murphy M.L., Roe B.B. (1975). A reporting system on patients evaluated for coronary artery disease. Report of the Ad Hoc Committee for Grading of Coronary Artery Disease, Council on Cardiovascular Surgery, American Heart Association. Circulation.

[16] Gülsün M.A., Funka-Lea G., Sharma P., Rapaka S., Zheng Y. Coronary centerline extraction via optimal flow paths and CNN path pruning. Proceedings of the Medical Image Computing and Computer-Assisted Intervention-MICCAI 2016: 19th International Conference.

[17] Xu L., He Y., Luo N., Guo N., Hong M., Jia X., Wang Z., Yang Z. (2021). Diagnostic Accuracy and Generalizability of a Deep Learning-Based Fully Automated Algorithm for Coronary Artery Stenosis Detection on CCTA: A Multi-Centre Registry Study. Front. Cardiovasc. Med..

[18] Griffin W.F., Choi A.D., Riess J.S., Marques H., Chang H.J., Choi J.H., Doh J.H., Her A.Y., Koo B.K., Nam C.W. (2023). AI Evaluation of Stenosis on Coronary CTA, Comparison with Quantitative Coronary Angiography and Fractional Flow Reserve: A CREDENCE Trial Substudy. JACC Cardiovasc. Imaging.

[19] U.S. Food and Drug Administration (FDA), Healthcare Products Regulatory Agency (2021). Good Machine Learning Practice for Medical Device Development: Guiding Principles.

[20] Chen C., Li X., Su Y., You Z., Wan R., Hong K. (2022). Adherence with cardiovascular medications and the outcomes in patients with coronary arterial disease: “Real-world” evidence. Clin. Cardiol..

[21] Naderi S.H., Bestwick J.P., Wald D.S. (2012). Adherence to drugs that prevent cardiovascular disease: Meta-analysis on 376,162 patients. Am. J. Med..

[22] Brown S.L., McRae D., Sheils E., McDonnell B.J., Khan I., James D.H. (2022). The effect of visual interventions on illness beliefs and medication adherence for chronic conditions: A scoping review of the literature and mapping to behaviour change techniques (BCTs). Res. Soc. Adm. Pharm..

[23] Feger S., Elzenbeck L., Rieckmann N., Marek A., Dreger H., Beling M., Zimmermann E., Rief M., Chow B.J.W., Maurovich-Horvath P. (2021). Effect of Computed Tomography Versus Invasive Coronary Angiography on Statin Adherence: A Randomized Controlled Trial. JACC Cardiovasc. Imaging.

[24] Han X., Luo N., Xu L., Cao J., Guo N., He Y., Hong M., Jia X., Wang Z., Yang Z. (2022). Artificial intelligence stenosis diagnosis in coronary CTA: Effect on the performance and consistency of readers with less cardiovascular experience. BMC Med. Imaging.

